# Germ cell-specific sustained activation of Wnt signalling perturbs spermatogenesis in aged mice, possibly through non-coding RNAs

**DOI:** 10.18632/oncotarget.13920

**Published:** 2016-12-15

**Authors:** Manish Kumar, Joshua Atkins, Murray Cairns, Ayesha Ali, Pradeep S. Tanwar

**Affiliations:** ^1^ School of Biomedical Sciences and Pharmacy, University of Newcastle, Callaghan, New South Wales, Australia

**Keywords:** Wnt βcatenin, spermatgonia, testicular cancer, fertility, Gerotarget

## Abstract

Dysregulated Wnt signalling is associated with human infertility and testicular cancer. However, the role of Wnt signalling in male germ cells remains poorly understood. In this study, we first confirmed the activity of Wnt signalling in mouse, dog and human testes. To determine the physiological importance of the Wnt pathway, we developed a mouse model with germ cell-specific constitutive activation of βcatenin. In young mutants, similar to controls, germ cell development was normal. However, with age, mutant testes showed defective spermatogenesis, progressive germ cell loss, and flawed meiotic entry of spermatogonial cells. Flow sorting confirmed reduced germ cell populations at the leptotene/zygotene stages of meiosis in mutant group. Using thymidine analogues-based DNA double labelling technique, we further established decline in germ cell proliferation and differentiation. Overactivation of Wnt/βcatenin signalling in a spermatogonial cell line resulted in reduced cell proliferation, viability and colony formation. RNA sequencing analysis of testes revealed significant alterations in the non-coding regions of mutant mouse genome. One of the novel non-coding RNAs was switched on in mutant testes compared to controls. QPCR analysis confirmed upregulation of this unique non-coding RNA in mutant testis. In summary, our results highlight the significance of Wnt signalling in male germ cells.

## INTRODUCTION

Spermatogonial stem cells (SSCs) sustain spermatogenesis throughout the life of a male by self-renewal and differentiation to committed progenitors [1]. These SSCs maintain testicular homoeostasis, and give rise to less differentiated spermatogonia which act as transit amplifying cells, thereby generating a large pool of cells which subsequently, in a series of steps, undergo terminal differentiation [1]. Signals deciding the fate of SSCs have been well-described in Drosophila [2], however, little is known about these signals in mammalian testis. Moreover, SSCs comprise only 0.02-0.03% of the testis and differentiate in a highly synchronized manner, consisting of mitotic expansion, meiotic divisions, and spermiogenesis. These stages are difficult to distinguish from committed progenitors via morphological analysis [1, 3]. Detailed analysis of SSC division is difficult due to lack of stage-specific markers. These factors make it hard to find cues deciding the fate of SSCs.

In seminiferous tubules, germ cells are organised in a highly orderly fashion where undifferentiated germ cells (SSCs and spermatogonia) are located at the periphery close to the basement membrane in the basal compartment and the differentiated germ cells are situated towards the lumen in the adluminal compartment. During spermatogenesis, spermatogonia undergo a series of developmental steps involving mitosis, meiosis and differentiation to produce round spermatids. These spermatids then transform to spermatozoa through a series of morphogenic events known as spermiogenesis [4]. Precisely balanced germ cell proliferation and differentiation is essential for the maintenance of homeostasis between different germ cell populations and any disruptions result in fertility defects and/or testicular cancer [5]. However, the mechanisms involved in dictating SSC's commitment to differentiation and spermatogenic progression remains undefined.

A number of evolutionarily conserved pathways are reported to be important for development, maintenance and differentiation of stem cells. These include Notch, the Transforming growth factor superfamily, Hedgehog, Fibroblast growth factor, and the Wnt signalling pathway [6]. Among these, the Wnt pathway has been reported as the most important pathway involved in the self-renewal and differentiation of stem cells in many different tissue types [7, 8]. Wnt signalling is essential for the development of primordial germ cells, which is the most primitive germ cell population [9]. Previous *in vitro* studies have shown involvement of the Wnt pathway in SSC homeostasis [10, 11]. Wnt signalling has been suggested to stimulate self-renewal of SSCs and proliferation of progenitor cell population [10, 11]. However, the precise role of Wnt/βcatenin signalling in germ cell development and differentiation in adult testis is currently unclear.

To infer the role of Wnt signalling in post-natal mammalian spermatogenesis, we first examined and detected active Wnt/βcatenin signalling in mouse, dog and human testes under normal physiological conditions. Using RNA and protein analysis, spermatgonial cell culture, thymidine analogues labelling, flow sorting, and a genetically modified mouse model, we have shown that overactivation of Wnt signalling in germ cells causes defects in proliferation and differentiation leading to premature loss of germ cells. Thus, our study has deciphered the precise role of Wnt signalling in germ cell development and differentiation.

## RESULTS

### Active Wnt signalling in testis of different mammalian species

**Figure 1:**
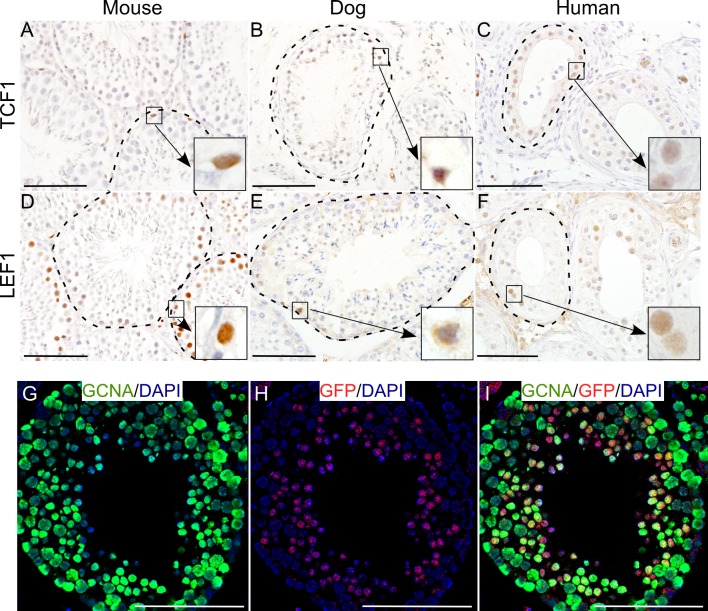
Wnt signalling activity in mammalian testis across the species **A.-F.** TCF1 (A-C) and LEF1 (D-F) expression (downstream targets of the Wnt pathway) in the seminiferous tubules of mouse, dog and human testis (N=5/each). **G.-I.** Nuclear GFP expression in GCNA positive-germ cells of TCFGFP mice marking active Wnt signalling. Nuclei are marked blue by DAPI. Bars: 100 μm.

The Wnt signalling pathway plays an important role in the development of mammalian gonads [12–14]. To ascertain the activity of Wnt signalling in testes of different mammalian species, we analyzed mouse, dog and human testes for the expression of well-established downstream targets, TCF1 (T-Cell Factor 1) and LEF1 (Lymphoid Enhancer-binding Factor 1), of this signalling pathway [13]. We found that across the species, testicular germ cells express TCF1 and LEF1 (Figure 1A-1F; N=5/each), suggesting that Wnt signalling is active during spermatogenesis in different mammalian species. We also examined testes from a well characterized Wnt reporter mouse model (TCFGFP, [15]). In this model, six copies of TCF/LEF responsive elements are placed upstream of the sequence coding for a fusion protein complex of Green Fluorescent Protein (GFP) and H2B histone protein, thereby expressing nuclear GFP in cells with active Wnt signaling [15]. Nuclear GFP expression was observed in the cells in seminiferous tubules (Figure 1H). Co-localization of GFP with GCNA (Germ Cell Nuclear Antigen; a germ cell marker) [13], confirmed that these GFP positive cells were indeed germ cells (Figure 1G-1I). These results confirm the activity of Wnt signalling in male germ cells of different mammalian species.

### Development of a mouse model with germ cell-specific constitutive activation of Wnt/βcatenin signalling

**Figure 2:**
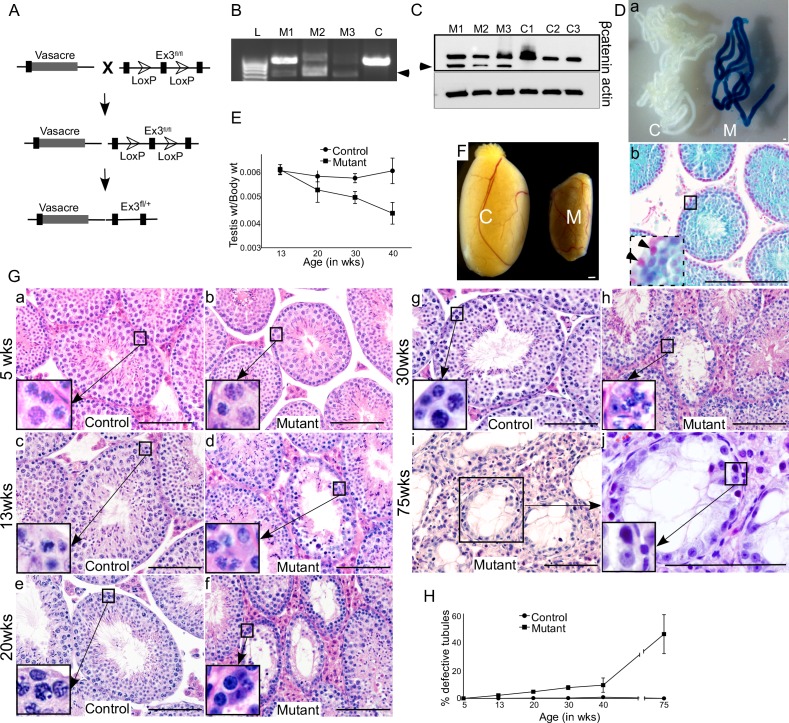
Overactivation of Wnt signalling in germ cells results in defective spermatogenesis **A.** Schematic representation of the mouse model. **B.** 700 bp amplified PCR product (marked by arrowhead), using DNA isolated from mutant testes (M1, M2, M3), confirming successful recombination of the βcatenin gene. Only wild-type (900 bp) band is present in control (C) group. **C.** Western blot analysis exhibiting a band in mutant testes corresponding to the truncated form of βcatenin (66 kDa), in addition to the band for wild type protein (96 kDa), which was found in both control and mutant testes. **Da.** Whole-mount X-Gal staining of the seminiferous tubules from control (C) and mutant (M) mice. Only seminiferous tubules from mutant mice exhibit lacZ expression. Db. lacZ expression was only present in germ cells (counterstained with Nuclear Fast Red). No lacZ expression was observed in Sertoli cells (inset, marked by arrowhead) indicating germ cell-specific cre activity. **E.** Testes weight/body weight ratio in mutant mice showing a progressive decline with age, as compared to control. **F.** Testis from 75 weeks old mutant mouse (M) exhibiting relatively smaller size compared to control (C). **G.** Histology of control and mutant testes exhibiting tubules with defective spermatogenesis in mutant mice. **H.** Graph depicting a progressive increase in percentage of defective tubules in mutant testes. Data are presented as mean ± SEM (N=3). Scale bars: 100 μm.

To study the role of Wnt/βcatenin signalling in germ cells, we developed a mouse model in which Vasa, a germ cell specific promoter, driven cre recombination removes floxed exon 3 sequence of the βcatenin gene, thereby resulting in constitutive activation of Wnt signalling specifically in germ cells (Vasacre;Ctnnb1fl(ex3/+); Figure 2A). Exon 3 of the βcatenin gene harbors the phosphorylation sites that are targeted by the Apc (Adenomatous polyposis coli) complex for its subsequent recognition by E3 ubiquitin ligase complex, and degradation by proteasome [16]. The deletion of exon 3, therefore, generates a stable and functional form of βcatenin protein, mimicking the activation of canonical Wnt signalling [16]. Successful recombination of the βcatenin gene was confirmed by polymerase chain reaction (PCR) using DNA isolated from mutant and control testes by presence of a 700 bp amplified PCR product (Figure 2B). Western blot analysis revealed a band in mutant testes corresponding to the truncated form of βcatenin (66 kDa), in addition to the band for wild type protein (96 kDa), which was found in both control and mutant testes. (Figure 2C). We detected both mutant and wild type protein bands in mutant testes because recombination of βcatenin allele only occurs in germ cells, but not in somatic cells (Leydig, Sertoli, peritubular, immune and endothelial cells). To confirm the germ cell specificity of Vasacre-mediated recombination, we mated Vasacre;Ctnnb1ex3/+ mice with ROSA26flGFP-NLS-lacZ reporter mice, in which lacZ expression is dependent on cre-mediated recombination. The resulting Vasacre;Ctnnb1ex3/+;lacZfl/+ mice showed lacZ expression specifically in germ cells (Figure 2D, a&b), but not in testicular somatic cells (Figure 2Db, inset). These results validate the specificity of our mouse model, in which successful cre-mediated recombination of βcatenin exon 3 allele results in constitutive activation of Wnt signalling in germ cells.

### Defective spermatogenesis in mutant mice

To study the effect of overactive Wnt signalling on germ cell development, we collected and analysed mutant (Vasacre;Ctnnb1fl(ex3/+)) and control testes at different developmental stages. There was a progressive reduction in testicular weight in mutant mice compared to controls (Figure 2E; N ≥ 3/each). Grossly, mutant testes were markedly smaller than control testes (Figure 2F, 75 weeks), which is indicative of defective spermatogenesis. Histological analysis at 5 weeks of age revealed no difference between control and mutant testes (N=3/each; Figure 2Ga and b). Both control and mutant testes had normal testicular architecture with a full complement of germ cells within seminiferous tubules (Figure 2Ga and b). However, at 13 weeks of age, germ cells were lost in some of the seminiferous tubules in mutant testes, but not in control testes (Figure 2Gc and d, and 2H). The number of defective seminiferous tubules with germ cell loss in mutant testes were increased progressively at 13, 20, 30, 40 and 75 weeks of age (Figure 2G and 2H; n ≥ 3/each). At 75 weeks of age, differentiated germ cells from more than 40% of seminiferous tubules were lost, and only a single layer of spermatgonial cells was present in the basal compartment of seminiferous tubules (Figure 2Gi and j). These results suggest that constitutive activation of Wnt/βcatenin signalling causes defects in germ cell development. Overactive Wnt signalling results in cancer development in various organ systems, such as ovary [17]. However, we did not find cancerous growth in testes of mutant or control mice (Figure 2G).

**Figure 3:**
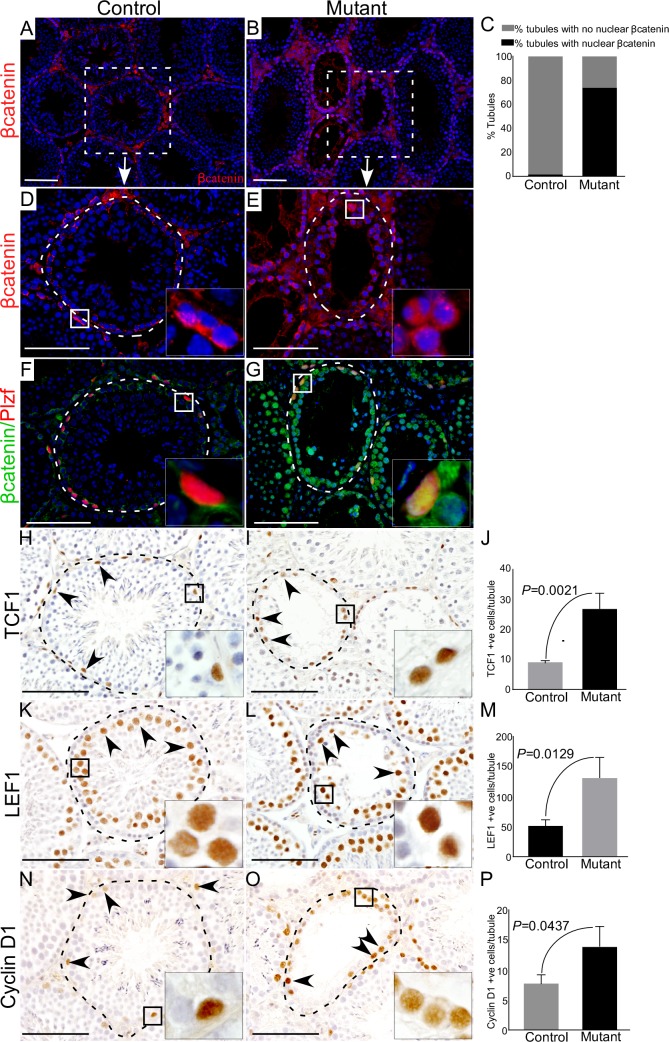
Altered expression of βcatenin and downstream targets of the Wnt pathway in germ cells of mutant mice **A.-E.** Immunostaining for βcatenin in control and mutant testes. **C.** Quantification of βcatenin expression manifesting 74% of seminiferous tubules with cytoplasmic and nuclear accumulation of βcatenin protein in mutant group while only 1.24% tubules with higher expression of βcatenin in control group. **F.** and **G.** Colocalization of βcatenin with Plzf (marks SSCs) depicting cytoplasmic and nuclear accumulation of βcatenin in Plzf positive cells of the mutant testis. **H.-P.** Quantification of expression of downstream targets of the Wnt signalling pathway (TCF1, LEF1 and Cyclin D1) demonstrating significantly higher expression in mutant mice as compared to controls. J, M and P represent the quantification graphs for the number of cells positive for TCF1 (J), LEF1 (M) and Cyclin D1 (P). Nuclei are marked blue by DAPI. Data are presented as mean ± SEM (N=3). Scale bars: 100 μm.

To confirm overactivation of Wnt/βcatenin signalling in mutant mice, we examined expression of βcatenin in control and mutant testes (Figure 3A-3E). In mutant testes, germ cells showed cytoplasmic and nuclear accumulation of βcatenin (Figure 3B, and 3E, N=3), whereas, mainly membranous expression of βcatenin was observed in control testes (Figure 3A and 3D, N=3). Histological examination of mutant testes revealed that spermatogonial cells were maintained in mutant mice at all the developmental stages examined in this study (Figure 2G). To rule out that spermatogonial cell are maintained due to the lack of recombination in this cell type, we determined βcatenin protein expression in spermatogonial cells by colocalising with a spermatogonial cell marker, Plzf (Promyelocytic leukaemia zinc finger protein) [18]. We found cytoplasmic and nuclear accumulation of βcatenin in Plzf-positive cells of mutant testes (Figure 3G), suggesting that hyperactivation of Wnt signalling also occurs in spermatgonial cells. Analysis of the key downstream targets of the Wnt pathway (TCF1, LEF1 and cyclin D1) showed a significant increase in the number of TCF1, LEF1, and Cyclin D1 positive germ cells in mutant testes compared to controls (Figure 3H-3P; N=3/each). Collectively, these results confirmed the abnormal accumulation of βcatenin in germ cells leads to defective spermatogenesis.

### Loss of differentiated germ cells (spermatocytes and spermatids) but not spermatogonial cells in mutant testes

**Figure 4:**
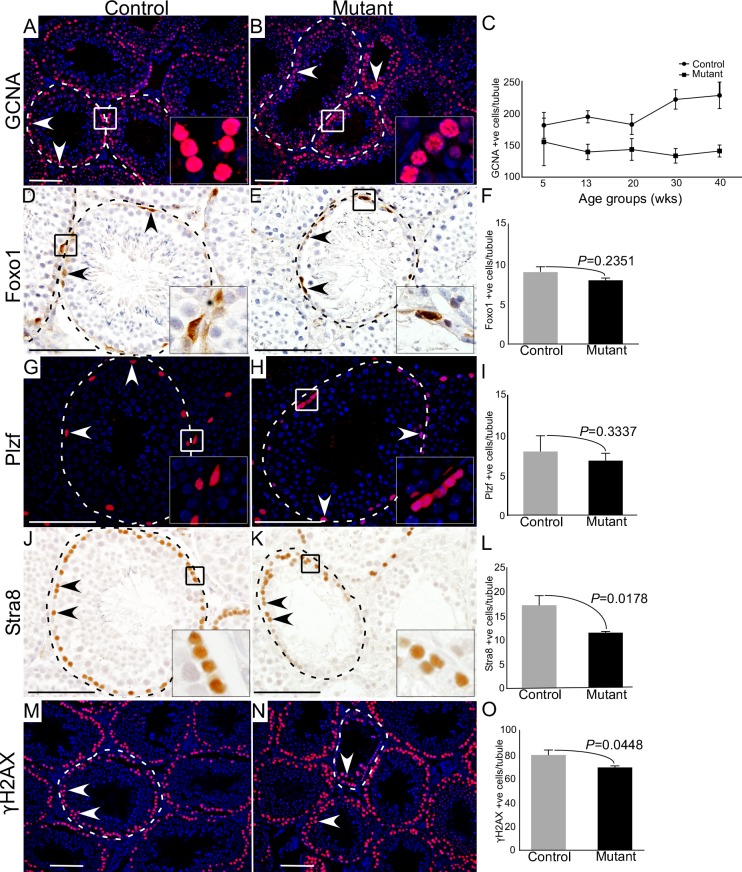
Hyperactive Wnt signalling results in reduced differentiation of germ cells **A.-C.** Reduced number of GCNA (a germ cell marker) positive cells are observed in mutant mice as compared to control group (N=3/each). Graphical representation of the number of GCNA positive cells at different ages in control and mutant mice. **D.-I.** Foxo1 and Plzf (markers for SSCs) positive cells in mutant animals compared to controls. **J.-L.** Significant decline in the number of Stra8 (marks spermatogonial cells committed for meiosis) positive cells in mutant testes with respect to control. **M.-O.** Number of γH2AX (marks meiotic germ cells) positive cells is significantly diminished in mutant testes. Nuclei are marked blue by DAPI. Data are presented as mean ± SEM (N=3). P value of <0.05 was considered significant. Scale bars: 100 μm.

To precisely determine the stages of germ cell development that are most affected by hyperactive of Wnt/βcatenin signalling, we compared expression of GCNA, which marks all the germ cells [13], Foxo1 (Forkhead box protein O1) and Plzf, two well-known markers of spermatogonial stem/progenitor cells [19], and Stra8 (Stimulated by retinoic acid 8), a marker for meiotically committed spermatogonial cells [20], in control and mutant testes (Figure 4; N = 3/each). We particularly examined mutant testes at 30 weeks of age, because both defective and normal seminiferous tubules were present in the same tissue section. We found a marked reduction in the number of GCNA positive cells per tubule in mutant testes compared to controls (Figure 4A-4C; N=3/each). However, the number of Foxo1 and Plzf positive cells were similar between control and mutant testes (Figure 4D-4I; N=3/each), suggesting that abnormal spermatogenesis in mutant mice might be due to defects in meiotic cells. Stra8-positive cells were significantly reduced in the mutant testes compared to controls (Figure 4J-4L; N=3/each). Next, we evaluated the expression of γ-histone 2AX (γH2AX), a well-established marker of meiotic germ cells [21], and found that γH2AX-postive cells were significantly reduced in mutant testes compared to respective controls (Figure 4M-4O; N=3/each). These findings suggested that constitutive activation of Wnt/βcatenin signalling in germ cells adversely affects meiotically committed progenitor cells and meiotic cells (spermatocytes and spermatids) without significantly influencing spermatogonial stem/progenitor cell population.

### Reduced germ cell proliferation in mutant testes

**Figure 5:**
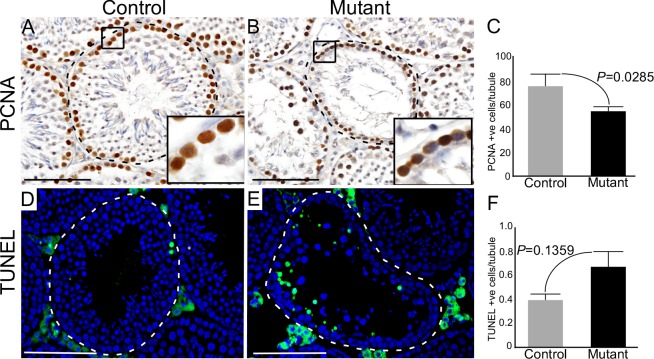
Sustained activation of Wnt signalling results in defective germ cell proliferation **A.-C.** Significant reduction in the number of PCNA (a marker for cell proliferation) positive cells in mutant mouse testes relative to controls. **D.-F.** TUNEL positive cells (a marker for cell death) in testes of both groups. Areas outline with squares in panel A and B are presented at a higher magnification in insets. Dotted lines outline seminiferous tubules. Nuclei are stained blue by DAPI. Data are presented as mean ± SEM (N=3). P value of <0.05 was considered significant. Scale bars: 100 μm.

Wnt signalling is a key regulator of cell proliferation and death [22]. To determine whether reduced germ cell proliferation or increased cell death are also responsible for the premature loss of germ cells in mutant testes, we analysed the expression of PCNA (Proliferating cell nuclear antigen; a marker for cell proliferation) [23], and TUNEL (Terminal deoxynucleotidyl transferase; a marker for cell death) [24] in mutant and control testes (Figure 5). In control testes, as expected, PCNA-positive cells were present only in the basal compartment of seminiferous tubules where mitotically active spermatogonial cells are normally located (Figure 5A; N=3). However, there was a significant reduction in the number of PCNA positive cells in mutant testes (Figure 5A-5C; N=3/each). No significant differences were observed in TUNEL positive cells between control and mutant testes (Figure 5D-5F; N=3/each), suggesting a limited contribution of germ cell death to the mutant phenotype.

**Figure 6:**
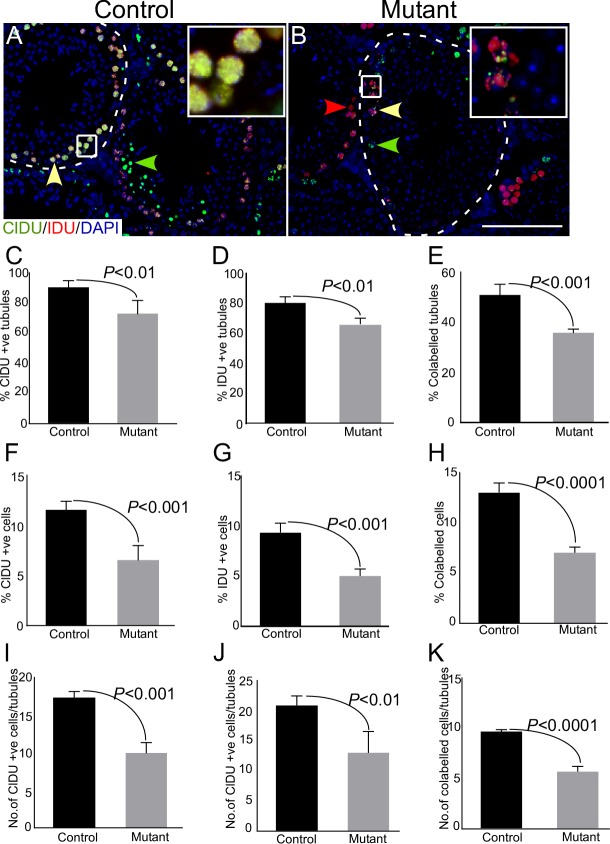
Thymidine analogue labelling unveils reduction in cellular turn over in mutant mice **A.-B.** Representative images of seminiferous tubules from control and mutant testes labelled by CldU followed by IdU at 5 days post-CldU administration. Areas outline with squares in panel A and B are presented at a higher magnification in insets. Dotted lines mark seminiferous tubules. **C.-K.** Reduced percentage of CldU+ve or IdU+ve or CldU+ve IdU+ve tubules in mutant testes. Percent of mono or colabelled cells and number of labelled cells/tubule also exhibit a decline in mutant group as compared to control. Nuclei are marked blue by DAPI. Data are presented as mean ± SEM (N=3). P value of <0.05 was considered significant. Scale bars: 100 μm.

In order to quantify, more accurately, the changes in proliferation of germ cells, we labelled germ cells sequentially with two different thymidine analogues (chloro-deoxyuridine; CldU and iodo-deoxyuridine; IdU) in both control and mutant mice. This method of sequential labelling enables identification of two or more successive rounds of cell division [25]. Since the doubling time of mouse spermatogonial cells is approximately 5 days [26], therefore, we administered both control and mutant mice sequentially with CldU followed by IdU, with a time-gap of 5 days to label the majority of cycling spermatogonial cells (Figure 6; N=3/each). Testes were collected 8 days post CldU injection (3 days-post IdU administration) because mouse spermatogenic cycle is of around 8 days in duration [27]. Immunofluorescence-based detection of CldU and IdU showed a decline in the number of seminiferous tubules with individually labelled as well as co-labelled germ cells in mutant testis (Figure 6C-6E). Furthermore, there was a reduction in the total number of labelled cells (Figure 6F-6H), as well as the number of labelled cells per tubule (Figure 6I-6K) in mutant testes as compared to controls. These results confirmed that germ cell proliferation was compromised upon sustained activation of Wnt/βcatenin signalling.

### Decreased post-leptotene germ cell population in mutant testes due to defective meiotic entry

**Figure 7:**
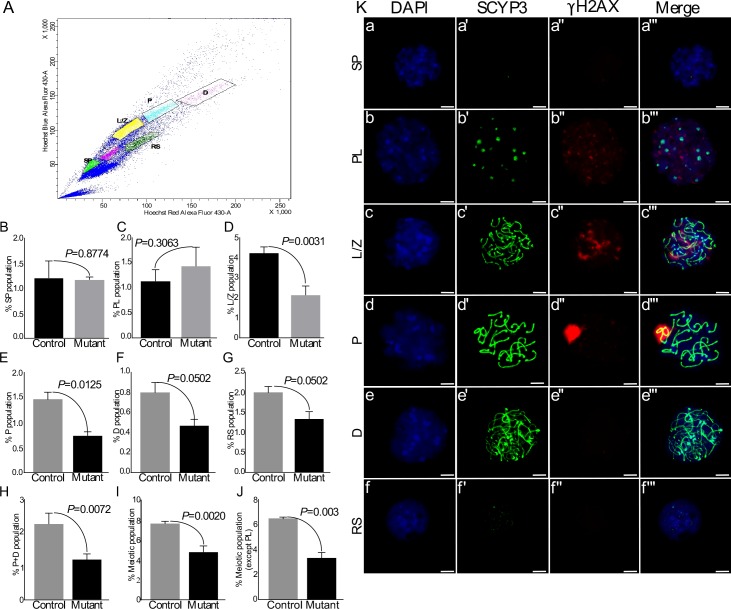
Flow sorting of germ cells shows reduction in meiotic germ cell population in mutant testes **A.** Representative scatter plot of different germ cell populations of a mouse testis. **B.-J.** No change in side population and Pre-leptotene germ cells. However, L/Z and subsequent meiotic populations are significantly reduced in mutant group. Total meiotic population was decreased in mutants. Ka-f SCYP3 and γH2AX double immunostaining on spread nuclei from flow sorted different testicular germ cell populations (SP, PL, L/Z, P, D, RS). Nuclei are stained blue by DAPI. SP: spermatogonia; PL: pre-leptotene; L/Z: leptotene/zygotene; D: diplotene; RS: round spermatids. Data are presented as means ± SEM (N=3). P value of <0.05 was considered significant. Scale bars: 5 μm.

The mammalian testis contains heterogeneous population of germ cells, including spermatogonial stem and progenitor cells, spermatocytes, and spermatids. SSCs both self-renew and generate a population of committed progenitor cells that later differentiate into meiotic spermatocytes, haploid spermatids, and spermatozoa [12, 13]. Our results so far have shown diminished proliferation in germ cells and reduction in the number of meiotically committed progenitor cells in mutant testes (Figure 4–6). To determine the specific stage of germ cell development affected by overactive Wnt/βcatenin signalling, we flow sorted different populations of testicular germ cells (N=3/each; Figure 7A). In agreement with the results of expression analysis (Figure 4), there was no difference in the number of spermatogonial stem/progenitor cells, as represented by the fraction of side population cells, between control and mutant testes (Figure 7B). Pre-leptotene (PL) is a stage before the actual start of meiosis that is characterised by physiological changes in the cytoplasm and nucleus, and marks the beginning of chromosomal condensation [28]. There was a non-significant increase in this population of germ cells (%PL) in mutant testes as compared to controls (Figure 7C). The beginning of meiosis is marked by Leptotene/Zygotene (L/Z) stage that is characterised by the assembly of synaptonemal complexes, and chromosomal pairing [28]. We found a significant reduction in %L/Z population in mutant testes as compared to controls (Figure 7D), indicating suppression at the beginning of meiotic division. Consistent with these results, there was a reduction in the percentage of cells at subsequent meiotic stages, which include pachytene (%P) identified by chromosomal crossover, diplotene (%D) marked by degradation of synaptonemal complexes and separation of homologous chromosomes, and round spermatids (Figure 7E-7G).

To ascertain the purity of sorted germ cell populations, we stained them with γH2AX and SCYP3 (a marker of synaptonemal complex assembly) [29] (Figure 7Ka-f’’’). The percent purity for different cell population was as follows: SP=94%, PL=86.53%, L/Z=96%, P=96%, D=72%, RS=86.67%. It was difficult to obtain a very pure %D population (72% purity) due to the presence of some pachytene stage spermatocytes. Therefore, the total of %P and %D populations was compared between control and mutant testes (Figure 7H). Similar to the individual results for %P and %D populations, a significant reduction was observed in the total of %P and %D populations of meiotic germ cells in mutant testes as compared to controls (Figure 7H). Moreover, the total meiotic population with (Figure 7I) and without (Figure 7J) %PL population was also reduced in mutant testis. These results revealed a stage specific brake in the meiotic progression of germ cells in mutant testis.

### Sustained activation of Wnt signalling does not affect regeneration potential of SSCs following chemical ablation of germ cells

**Figure 8:**
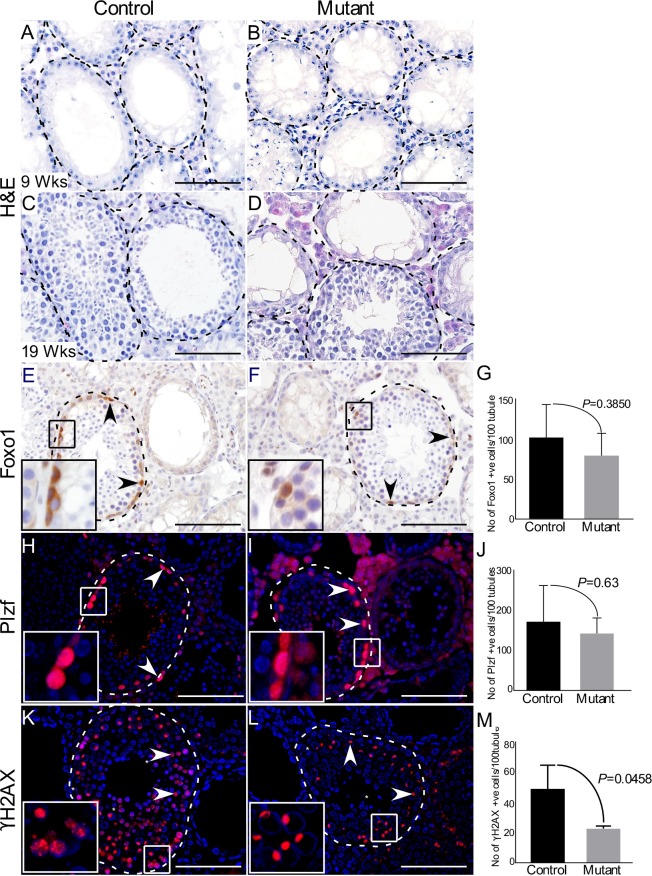
Active Wnt signalling does not affect regenerative potential of spermatgonial cells **A.-B.** Complete loss of germ cells 4 weeks after busulfan administration in both control and mutant mice. **C.-D.** Histological analysis of testes showed recovery of spermatogenesis in some tubules at 19 weeks. **E.-J.** No change in Foxo1 and Plzf-positive spermatgonial stem/progenitor cells in both groups. **K.-M.** Mutant testis shows reduced number of γH2AX-positive meiotic cells as compared to controls. Areas outline with squares in panel E, F, H, I, K and L are presented at a higher magnification in insets. Dotted lines outline seminiferous tubules. Nuclei are marked blue by DAPI. Data are presented as mean ± SEM (N=3/each). P value of <0.05 was considered significant. Scale bars: 100 μm.

To examine if overactivation of Wnt/βcatenin signalling has any effect on the regenerative potential of SSCs, we treated 5 weeks old control and mutant mice with busulfan (N=3/each). Busulfan has been used in previous studies for ablation of germ cells to study the regenerative potential of SSCs [30, 31]. As expected, a complete loss of germ cells was observed in control and mutant testes, 4 weeks post-treatment (Figure 8A and 8B). After 14 weeks of the treatment, histological analysis revealed that spermatogenesis has recovered in many tubules in both control and mutant testes with no apparent difference in the rate of germ cell repopulation (Figure 8C and 8D). Immunolabelling with germ cell markers showed no significant differences in spermatogonial stem/progenitor cells (Foxo1 and Plzf) between two groups (Figure 8E-8J). However, consistent with previous results (Figure 4M-4O), a significant reduction in meiotic germ cell population (γH2AX) was observed (Figure 8K-8M). These data demonstrated that overactive Wnt signalling causes no obvious perturbations in repopulating potential of spermatgonial stem cells.

### In vitro model of spermatogonial cells confirmed reduced cell proliferation and viability due to overactivation of Wnt signalling

**Figure 9:**
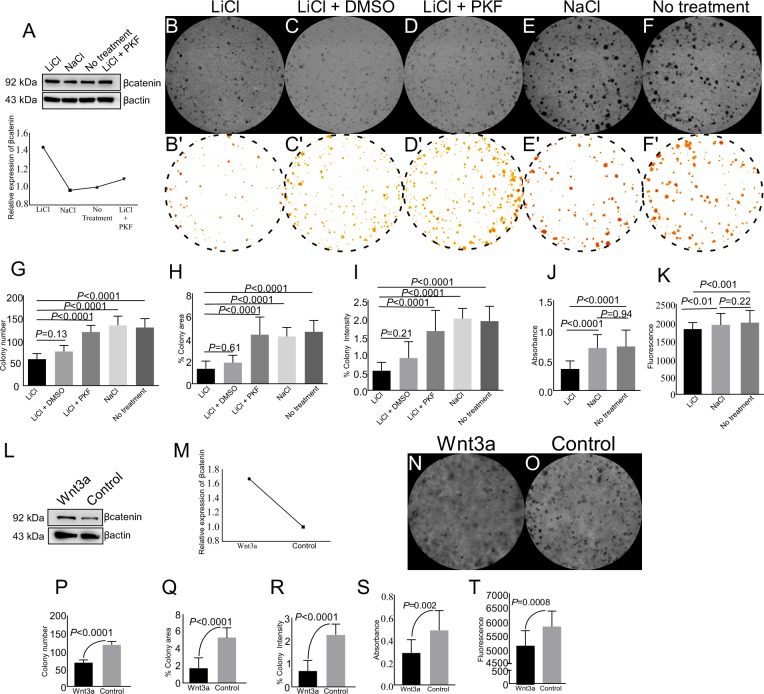
Pharmacological activation of Wnt signalling reduces colony formation, cell viability, and cell proliferation of spermatogonial cells **A.** Western blot analysis showed enhanced expression of βcatenin in GC1 cells with LiCl treatment. **B.-I.** LiCl treatment reduced colony number, colony area, and colony staining intensity as compared to NaCl and no treatment group. Images presented in panel B’-F’ are used for quantification from panel **B-F.** (A-I) The presence of 0.125 μM PKF118-310 (a Wnt inhibitor) rescued the effect of LiCl by decreasing the levels of βcatenin protein. **J.-K.** Overactive Wnt signalling reduced proliferation and viability of GC1 cells. **L.-M.** Increased expression of βcatenin in GC1 cells cultured in presence of Wnt3a condition media. **N.-T.** Wnt3a treatment results in reduced colony number, colony area, colony intensity, cell proliferation, and cell viability as compared to control group (O). Data are representative of at least three independent experiments and are expressed as the mean ± SEM. P value of <0.05 was considered significant.

In order to manipulate Wnt signalling more precisely without the influence of paracrine signals that are operational in vivo, we utilized a well-studied *in vitro* model of spermatogonial cells (GC1 cells, [32]) to investigate effects of overactive Wnt signalling on germ cells. GC1 cells were cultured in presence of 5 mM LiCl (Lithium Chloride), a well-known activator of Wnt signalling [33]. LiCl treated GC1 cells showed 1.4-fold increase in βcatenin expression as compared to control (NaCl) (Figure 9A), thereby, confirming hyperactive Wnt signalling in these cells. There was a significant reduction in the number of colonies formed, colony area and colony staining intensity in LiCl treated cells as compared to control groups (Figure 9B, 9E-9F and 9G-9I). In order to confirm that the reduction is mediated through Wnt signalling, we treated these cells with both LiCl and PKF118-310, a potent inhibitor of Wnt signalling pathway [34]. Western blot analysis confirmed that βcatenin protein levels were decreased in the presence of PKF118-310, and were similar to NaCl treated group (Figure 9A). A similar trend was observed in LEF1 protein levels with LiCl and/or PKF118-310 treatments (SFigure 1). The addition of PKF118-310 diminished the effect of LiCl on colony number, area and staining intensity (Figure 9C and 9D, 9G-9I). Similar to our in vivo results, activation of Wnt signalling with LiCl treatment reduced cell viability and cell proliferation as compared to controls (Figure 9J and 9K).

LiCl activates Wnt signalling by inhibiting of GSK3β [35]. However, GSK3β is also involved in other signalling pathways, such as the PI3K pathway [36]. To confirm that reduced cell proliferation and viability of LiCl treated GC1 cells is specifically mediated through Wnt signalling, we cultured GC1 cells in Wnt3a conditioned media or control media for 5 days, and proteins were isolated from these cells. Western blot analysis revealed 1.7 fold increase in βcatenin expression in GC1 cells cultured with Wnt3a conditioned media, relative to controls (Figure 9L and 9M). Similar to LiCl treatment, Wnt3a conditioned media treatment also led to reduction in colony number, colony area, and colony staining intensity (Figure 9N-9R). Cell viability and proliferation of GC1 cells was also decreased in Wnt3a conditioned media treated group (Figure 9S and 9T). Collectively, these results showed overactive Wnt/βcatenin signalling negatively regulate spermatgonial cell proliferation and viability.

### Abnormal expression of non-coding RNAs in mutant testis

**Figure 10:**
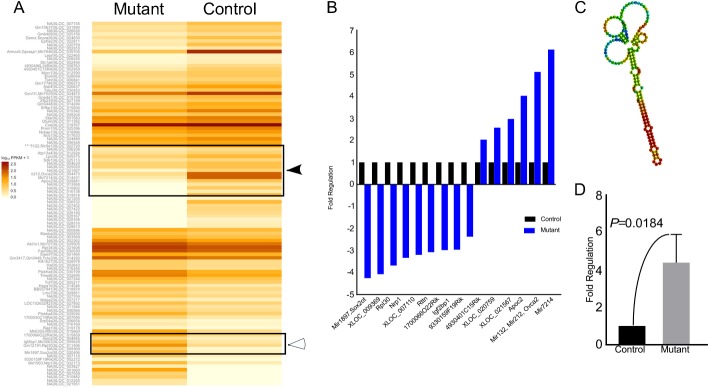
Altered expression of non-coding RNAs in mutant testes **A.** Heat-map from RNA sequencing of whole testes of control and mutant mice. The log2 FPKM (Fragments Per Kilobase of transcript per Million mapped reads) values for 104 genes are used for generation of the heat map in two groups. The colours correspond to the log2 of FPKM values, ranging from bright brown (2.5) to white (zero). Solid arrowhead marks genes that are turned off in mutant condition while empty arrowhead marks genes turned on in mutant condition. **B.** Graphical representation of the genes showing at least two-fold change in mutant testes. **C.** Putative structure of a novel non-coding RNA switched on in mutant mouse testes. **D.** Four fold up-regulation of expression of the novel non-coding RNA in the mutant testis using qRT-PCR analysis. Data is presented as means ± SEM (N=3/each).

To identify the probable targets of the Wnt signalling pathway responsible for pathological changes in mutant testes, we performed RNA sequencing analysis of control and mutant testes (N=3/each). We were able to identify 20 genes of unknown functions, 15 of which were switched off (solid arrow head), and 5 were switched on (hollow arrowhead) in mutant testes (Figure 10A). 15 genes showed a minimum of two fold change in expression between control and mutant group (Figure 10B). We also found a novel non-coding RNA at chromosome-1, loci:19037508-19037682 with FPKM (Fragments Per Kilobase of transcript per Million mapped reads) value of 21.13 in mutant testes, and zero case value in control group (Figure 10C). This was further confirmed by quantitative PCR, which revealed 4-fold up-regulation of this non-coding RNA (Figure 9D) in mutant testes. These results suggest that Wnt signalling regulates spermatogenesis possibly through non-coding RNAs.

## DISCUSSION

In this study, we have developed a mouse model with germ cell specific overactivation of Wnt signalling, and demonstrated the requirement of this pathway for the proliferation and differentiation of germ cells in mammalian testis, which was also confirmed using an *in vitro* cell culture system. We flow sorted different germ cell populations, and were able to trace overactive Wnt signalling mediated differentiation defects at the leptotene/zygotene stage of meiosis, suggesting that this specific stage is an important check point in the process of germ cell development (Figure 7). Our results have highlighted the requirement of balanced Wnt signalling in mammalian spermatogenesis.

In recent years, few studies have contributed towards understanding the role of Wnt signalling in male germ cell biology. However, insufficient data is available regarding the functional relevance of this signalling pathway in male germ cell development and differentiation because these studies have reported many divergent phenotypes probably due the use of different Cre mice. For example, the loss of βcatenin in testicular germ cells using Stra8-icre results in no defects in germ cell development, sperm production, and fertility [37], suggesting Wnt/βcatenin is dispensable for male germ cell development and fertility. In contrast, ablation of βcatenin in germ cells using Axin2cre and Protamine 1-cre leads to premature germ cell loss, reduced sperm count and infertility [38, 39]. Similarly, alterations in Wnt signalling using Cytochrome P4501A1cre/Ahcre causes disruption of spermatogenesis and loss of germ cells [40]. Unintended cre recombination in other testicular cell types, such as somatic cells, has been proposed as one of the reasons for the differences in testicular phenotypes reported in these studies [37]. For example, Protamine 1-cre and Ahcre, in addition to germ cells, also induce recombination of flox alleles in the somatic cells of testis [37, 40]. It is already well established that balanced Wnt/βcatenin signalling in testicular somatic cells is essential for male germ development and fertility, and any aberrations in this signalling pathway in these cells cause severe defects in germ cell development and spermatogenesis [12, 13, 41–43]. Therefore, it is unclear whether previously reported phenotypes in germ cells are due the defective Wnt signalling in testicular germ and/or somatic cells. Our study is the first to use the germ cell-specific cre, Vasacre, model to provide evidence for the role of Wnt signalling in germ cell biology.

In seminiferous tubular environment, undifferentiated spermatogonia proliferate and differentiate to constitute a heterogeneous population of germ cells that can be divided into two pools, spermatogonial stem/progenitor population and differentiated spermatocytes [1]. A fine balance between these two populations is essential to maintain testicular homeostasis and deviations result in testicular cancer or infertility [5]. Wnt signalling is a known driver of self-renewal and differentiation of adult stem cells [44]. Sertoli cells secrete Wnt6 that acts on SSCs leading to the activation of Wnt pathway which then regulates proliferation of undifferentiated spermatogonia [38]. Using marker analysis and sequential DNA double labelling technique, we have shown that sustained Wnt activity results in reduced germ cell proliferation. Moreover, DNA double labelling has shown increased refractory period between two successive germ cell divisions (indicated by reduced co-labelled cells in mutant testes), leading to diminished capacity of individual germ cell to contribute towards the germ cell pool. Unchanged spermatogonial stem/progenitor population with decreased germ cell proliferation raises the possibility of reduced generation of progenitor population committed for differentiation. Marker analysis (Stra8 and γH2AX) and FACS results confirmed reduced germ cell differentiation. Therefore, our results demonstrate that overactivation of Wnt pathway causes premature germ cell loss by affecting their proliferation and differentiation.

Most of the studies focusing on the role of Wnt pathway in SSCs have drawn conclusions from results based on mRNA expression profile and immunofluorescence or histology. Little has been done to understand the mechanisms underlying Wnt signalling mediated regulation of spermatogenesis. Towards this end, we sequenced RNA isolated from whole testis of control and mutant mice, and found differential expression of many genes. Some of the non-coding RNAs were completely switched off while some were specifically switched on in the knockout condition only. Non-coding RNAs plays critical roles in transcriptional and posttranscriptional gene regulation and also affects mRNA expression [45]. One example of such non-coding RNAs is mhrl (meiotic recombination hot spot locus) which negatively regulates Wnt signalling pathway through its protein partner Ddx5/p68 [46]. The functions of non-coding RNAs reported by our study have not yet been defined in the literature. Currently, we are investigating the functions performed by these non-coding RNAs, and are developing assays to validate the results. In future studies, we seek to look into the functional relevance of these non-coding RNAs and other differentially expressed genes in the regulation of spermatogenesis.

In conclusion, our work using *in vitro* and *in vivo* model systems showed that aberrations in Wnt signalling leads to defective spermatogenesis primarily due to the abnormalities in meiotically committed germ cells. As abnormal Wnt signalling is associated with human male infertility and testicular cancer, it is likely that similar mechanisms might be operational in human patients.

## MATERIALS AND METHODS

### Mouse breeding and husbandry

Mice used in this study were maintained on C57BL/6;129SvEv mixed genetic background and were kept under standard animal housing conditions. All the experimental procedures undertaken on mice were approved by the Animal Care and Ethics Committee, University of Newcastle. For animal care and experimental procedures guidelines of, New South Wales Animal Research Act, New South Wales Animal Research Regulation, and the Australian code for the care and use of animals for scientific purposes guidelines were followed. For developing a mouse model (Vasacre;Ctnnb1ex3/+) with germ cell specific overactivation of Wnt/βcatenin signalling, Tg(Ddx4-cre)1Dcas/J (Vasacre) mice [47] were crossed with Ctnnb1tm1Mmt [16]. Vasacre;Ctnnb1ex3/+ mice were crossed with homozygous ROSA26flGFP-NLS-lacZ mice [48] to develop Vasacre;Ctnnb1ex3/+;lacZfl/+. REDExtract-N-Amp™ Tissue PCR Kit (Sigma, MO, USA) was used for DNA isolation. Primers used for genotyping are listed in Table 1. Dog testicular tissue samples were collected at the department of Veterinary Pathology, University of Sydney.

**Table 1:** List of Primers used for genotyping

Transgene	Forward Primer	Reverse Primer
Vasacre	5’CACGTGCAGCCGTTTAAGCCGCGT3’	5’TTCCCATTCTAAACAACACCCTGAA3’
Ctnnb1tm1Mmt	5’GACACCGCTGCGTGGACAATGA3’	5’GTGGCTGACAGCAGCTTTTCTA3’
ROSA26flGFP-NLS-lacZ	5’AAAGTCGCTCTGAGTTGTTAT3’	5’TCCAGTTCAACATCAGCCGCTACA3’
Novel non-coding RNA	5’CCACATAGAAATACTCTGCTCTC3’	5’TAAGGCTCTGTAACCCTCAT3’
βactin	5’TGTTACCAACTGGGACGACA3’	5’GGGGTGTTGAAGGTCTCAAA3’

### Germ cell ablation model

A minimum of three mice from each control and mutant group (5 weeks old) were given a single intraperitoneal injection of Busulfan (10 mg/ml in a 1:1 mixture of dimethyl sulfoxide and distilled water; @ 40 mg/Kg body weight). The animals were sacrificed 14 weeks post-injection and testes were collected. The testes were then fixed overnight in 4% paraformaldehyde (PFA, Electron Microscopy Sciences, PA, USA) at 4°C. Until processing, the tissues were stored in 70% Ethanol at 4°C.

### Thymidine analogue labelling

Control and mutant mice (N=3/each) were administered with CldU and IdU (10 mg/ml) by intraperitoneal injections (100 μg/g body weight) at 30 weeks of age. For labelling, first CldU was injected followed by IdU with a gap of 5 days. The animals were sacrificed and tissues were collected 3 days after IdU administration. The tissues were then fixed overnight at 4°C in 4% PFA.

### Histology, immunohistochemistry/immuno-fluorescence and TUNEL assay

Histology and immunohistochemical analysis were done as described by us in [12]. Briefly, tissues were overnight fixed at 4°C, in 4% PFA, embedded in paraffin and 5 μm sections were prepared. Slides were deparaffinised and incubated with primary antibodies including βcatenin (610154, BD Transduction Labs, CA, USA); PCNA, Plzf (sc-56: PCNA; sc-22839: Plzf; Santa Cruz Biotechnology, CA, USA); Foxo1, LEF1, TCF1 (#2880: Foxo1; #2230: LEF1; #2203: TCF1; Cell Signaling Technology, MA, USA); Cyclin D1, SCYP3, Stra8 (ab16663: Cyclin D1; ab15093: SCYP3; ab49602: Stra8; Abcam, Vic, Australia); GCNA [49]; γH2AX (#05-636: γH2AX; Millipore, MA, USA), αSMA (c6198, Sigma, MO, USA) and AlexaFluor secondary antibodies (1:250; Jackson ImmunoResearch Labs, PA, USA). For detection of apoptotic cells, TUNEL assay was performed on paraffin sections as per the instructions provided with the kit (Millipore). For cell counting images at 20x magnification were taken with Olympus DP72 microscope keeping same exposure and gain for both control and mutant tissues. Each testis was divided into four sections and at least two images were randomly selected from each section from minimum three control and mutant animals. The cells were counted using ImageJ (National Institute of Health, USA).

### Flow sorting of germ cells

Digestion of testes and flow sorting of germ cells were performed as described in [29] with minor modifications. Briefly, testes from 30 weeks old control and mutant mice (N=3/group) were decapsulated and gently washed with DMEM High Glucose (HyClone, GE Life Sciences) to remove interstitial cells. Seminiferous tubules were collected and digested with collagenase (1 mg/ml) and DNase (5 units, Promega) for 15 minutes. Tubules were removed from collagenase and digested with 0.25% trypsin/EDTA and DNase (5 units) for 15 minutes. Tissue digestion was then stopped by adding foetal bovine serum (FBS). The digested cell suspension was filtered through 40 μm tissue filter (BD Biosciences). The cells were given one wash with DMEM and 1×106 cells/ml suspension was prepared. The cells were incubated with Hoechst 33342 (1:5000; 10 mg/ml stock; Sigma) at 25°C for 1 hour. The Hoechst 33342 labelled cells were again filtered with 40 μm tissue filter. Propidium Iodide was added to distinguish live and dead cells (1:2000; 10 mg/ml; Sigma) and the cells were sorted using FACSDiva (version 6.1.3).

### Meiotic spread and SCYP3/Plzf immunostaining

Meiotic spreads were prepared as described in a previous report [29]. Briefly, 50 μl of flow sorted cell suspension was mixed with equal volume of hypotonic extraction buffer (30 mM Tris, 50 mM Sucrose, 17 mM trisodium citrate dihydrate, 5 mM EDTA in water) for 30 minutes at room temperature. Cells were pelleted at 5000 g, 4 minutes and resuspended in 100 mM Sucrose. The cell suspension was then applied on Poly-L-Lysine coated cover glass slides containing 1% PFA/0.15% Triton X-100 and spread using coverslip. These slides were then dried overnight in humidified chamber at RT and immersed into 0.4% Photoflo and dried at RT. These slides can be stored at -20°C.

For immunostaining, frozen slides were thawed and washed serially for 5 minutes each with PBS containing 0.5% and 0.05% Triton X-100, followed by a 5 minutes wash with PBS only. Slides were blocked with blocking buffer (3% BSA, 0.15% Triton X-100 in PBS) for 30 minutes at RT followed by overnight incubation in γH2AX (1:1000) and SCYP3 (1:750) primary antibodies at 4°C. AlexaFluor secondary antibodies (1:250; Jackson ImmunoResearch Labs) were used for signal detection. Images were taken with Olympus FV1000 confocal microscope (Olympus, Japan).

### Cell culture

GC1 cells were cultured in Dulbecco's modified Eagle's medium (HyClone, GE Life Sciences), supplemented with 10% FBS (Interpath, Vic, Australia), 1% L-Glutamine (Sigma), 1% Penicillin-Streptomycin (Lonza, Vic, Australia) and 1% Sodium pyruvate (HyClone, GE Life Sciences) at 37°C in a humidified, 5% CO_2_ incubator. These cells were treated with 5 mM LiCl (a Wnt activator, [32]), 5 mM NaCl and 0.125 μM PKF118-310 (a Wnt inhibitor, [34]). For immunostaining of LEF1, GC1 cells were cultured on Poly-L-Lysine coated cover glass slides and treated as above. Cell viability assay was performed using Vision Blue Assay kit (Biovision) and cell proliferation assay was done using WST assay (Sigma), as per manufacturer's instructions. Colony formation assay (CFA) was performed and analysed as described by Guzman et al [50]. Wnt3a and Lcell were purchased from the American Type Culture Collection (ATCC). Conditioned media were prepared as mentioned in [51].

### Western blot analysis

Proteins were extracted from 5 weeks old mouse testes and from GC1 cells using ice-cold radioimmunoprecipitation assay buffer (RIPA) supplemented with protease and phosphatase inhibitors. Equal amounts of protein were loaded and resolved by 10% SDS-PAGE gel, and transferred to nitrocellulose membrane. The membrane was blocked in 5% milk (w/v) in Tris-buffered saline (0.1% Tween-20) for 1 hour at room temperature followed by overnight incubation at 4°C with primary antibodies (βcatenin 1:2000, LEF1 1:1000 in 2.5% w/v BSA, 1x TBS, 0.1% Tween-20; #8480 and #2230: Cell Signalling Technologies; βactin as loading control 1:5000 in 2.5% w/v BSA, 1x TBS, 0.1% Tween-20; JLA20: DSHB). Membranes were developed following 1 hour incubation with horseradish peroxidase-conjugated secondary antibodies (Jackson ImmunoResearch, West Grove, PA). Densitometric analysis was performed using ImageJ (National Institute of Health, USA).

### RNA isolation and sequencing

Total RNA was isolated from 30 weeks old control and mutant mice (N=3/each) using RNeasy® Mini kit (Qiagen, Vic, Australia) following manufacturer's instructions. RNA sequencing was performed at the Australian Genome Research Facility (AGRF, Brisbane, QLD, Australia) using Illumina platform (Illumina Inc. San Diego, CA, USA). Illumina HiSeq 2000 RNA-seq sequence of a 50 bp single end run were produced and libraries were prepared using Illumina TrueSeq stranded protocols. Image analysis was performed in real time by the HiSeq Control Software (HCS) v1.4.8 and Real Time Analysis (RTA) v1.18.61. RTA performs real-time base calling on the HiSeq instrument computer. The Illumina CASAVA 1.8.2 pipeline was used to generate the sequence data in a standard FASTQ format. Per base sequence quality for all the samples were analysed and values above Q30 were considered excellent. The cleaned sequence reads were then aligned against the Mus musculus genome (Build version mm10). The Tophat aligner (v1.3.1) was used to map reads to the genomic sequences. The transcripts were assembled utilizing reference based annotation with the Cufflinks tool (v2.1.1) using the reference annotation based assembly option (RABT) generating assembly for known and potentially novel transcripts.

### Quantitative real-time polymerase-chain reaction (qRT-PCR)

RNA isolated from 30 weeks old control and mutant testes (N=3/each), as described above, were used for analysis of novel non-coding RNA. RNA (1 μg) was added as a template to reverse-transcriptase reactions carried out using RT^2^ First Strand Kit (Qiagen). qRT-PCR were carried out with the resulting cDNAs in triplicate using SYBR Green ROX qPCR Mastermix (Qiagen) and ABI 7900 HT FAST (Applied Biosystems). Experimental Ct values were normalized to βactin and relative RNA expression in mutant versus control animal was calculated. Specificity of primer binding only to non-coding RNA of interest was confirmed by presence of a single band of 101 base pairs in PCR. Sequences of primers used are mentioned in Table 1.

### Statistical analysis

For every experiment, data were collected from a minimum of three repeats or three animals from control and mutant group. Data were analysed and graphed with GraphPad Prism 6.0. Student's t tests were used to calculate significance and P value of <0.05 was considered significant. Data are expressed as mean ± SEM.

## SUPPLEMENTARY MATERIALS FIGURE


