# Supplementary material for: Germ cell-specific sustained activation of Wnt signalling perturbs spermatogenesis in aged mice, possibly through non-coding RNAs
Source: Oncotarget. 2016 Dec 15;7(52):85709–27. doi: 10.18632/oncotarget.13920 (PMC5349868; doi:10.18632/oncotarget.13920)
Supplement: Supplementary file 1 [file oncotarget-07-85709-s001.pdf]

Germ cell-specific sustained activation of Wnt signalling perturbs spermatogenesis in aged mice, possibly through non-coding RNAs

Supplementary Material

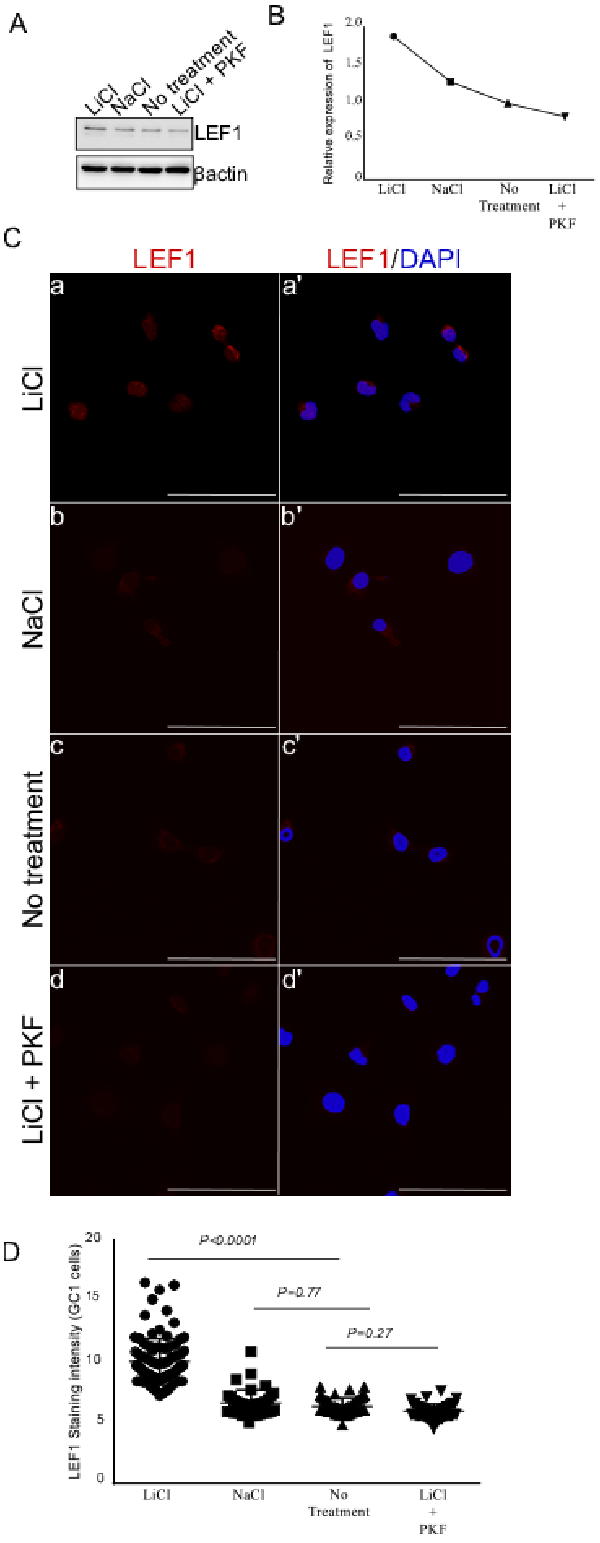

SFig. 1. Compared to controls, LiCl treatment increases and PKF treatment decreases the expression of LEF1 protein in GC1 cells.
